# Draft Genome Sequence of *Bacillus shackletonii* LMG 18435^T^, Isolated from Volcanic Mossy Soil

**DOI:** 10.1128/genomeA.01689-15

**Published:** 2016-02-04

**Authors:** Jie-ping Wang, Bo Liu, Guo-hong Liu, Ci-bin Ge, Rong-feng Xiao, Xue-fang Zheng, Huai Shi

**Affiliations:** Agricultural Bio-Resources Research Institute, Fujian Academy of Agricultural Sciences, Fuzhou, Fujian, China

## Abstract

*Bacillus shackletonii* LMG 18435^T^ is a Gram-positive, aerobic, and spore-forming bacterium. Here, we report the 5.30-Mb draft genome sequence of *B. shackletonii* LMG 18435^T^, which will promote its fundamental research and provide useful information for genomic taxonomy and phylogenomics of *Bacillus*-like bacteria.

## GENOME ANNOUNCEMENT

The type strain LMG 18435^T^ (=CIP 107762^T^) of *Bacillus shackletonii* was isolated from mossy soil taken from the eastern lava flow of northern Candlemas Island, South Sandwich archipelago (1). Recently, the thermophilic strain K5 of *B. shackletonii* was isolated from a biotrickling filter and appeared to have a strong capacity for producing polyhydroxybutyrate (up to 2.28 g/L) (2). Because of the application prospects and lack of genomic information of *B. shackletonii*, its type strain LMG 18435^T^ was selected as one of the research objects in our genome sequencing project for genomic taxonomy and phylogenomics of *Bacillus*-like bacteria. Here, we present the first draft genome sequence of *B. shackletonii*.

The genome sequence of *B. shackletonii* LMG 18435^T^ was obtained by paired-end sequencing on the Illumina HiSeq 2500 system. Two DNA libraries with insert sizes of 272 and 5,106 bp were constructed and sequenced. After filtering of the 1.29-Gb raw data, the 1.23-Gb clean data were obtained, providing approximately 233-fold coverage. The reads were assembled via the SOAPdenovo software version 1.05 (3), using a key parameter K setting at 76. Through the data assembly, 24 scaffolds with a total length of 5,297,592 bp were obtained, and the scaffold *N*_50_ was 3,831,633 bp. The average length of the scaffolds was 220,733 bp, and the longest and shortest scaffolds were 3,831,633 bp and 536 bp, respectively. A total of 95.35% clean reads could be aligned back to the genome, which covered 99.36% of the sequence.

The annotation of the genome was performed using the NCBI Prokaryotic Genomes Automatic Annotation Pipeline (PGAAP) (http://www.ncbi.nlm.nih.gov/genome/annotation_prok) utilizing GeneMark, Glimmer, and tRNAscan-SE tools (4). A total of 5,522 genes were predicted, including 5,401 coding sequences, 94 tRNAs, and 27 rRNAs. There were 3,666 and 2,210 genes assigned to the COG and KEGG databases, respectively. The average DNA G+C content was 36.71%, agreeing with the value 35.4 mol% (1).

### Nucleotide sequence accession numbers.

This whole-genome shotgun project has been deposited at DDBJ/EMBL/GenBank under the accession number LJJC00000000. The version described in this paper is the first version, LJJC01000000.
